# Activated Partial Thromboplastin Time and Mortality in Coronary Artery Bypass Grafting Patients

**DOI:** 10.1155/2022/2918654

**Published:** 2022-09-17

**Authors:** HuanRui Zhang, Wen Tian, Guoxian Qi, Longfeng Sun, Xiufang Wei

**Affiliations:** Department of Geriatric Cardiology, The First Hospital of China Medical University, China

## Abstract

**Background:**

To evaluate the prognostic value of preoperative activated partial thromboplastin time (APTT) in patients who underwent coronary artery bypass grafting (CABG).

**Methods:**

All data were extracted from the Medical Information Mart for Intensive Care III (MIMIC-III) database. The study population was divided to two groups according to the optimal cut-off value of APTT calculated by X-tile software, and Cox proportional hazard model was used to define independent effect of APTT on 4-year mortality. Survival curves were estimated by the Kaplan-Meier method, and the area under the receiver-operating characteristic curve (AUC) was calculated to compare APTT with other severity scores. Propensity score matching (PSM) analysis were applied to ensure the robustness of this study.

**Results:**

A total of 2,706 patients were included. The optimal cut-off value of APTT for 4-year mortality was 44 seconds. The Cox proportional hazard model showed that patients with APTT ≥ 44 had a significantly higher risk of all-cause death than those with APTT < 44 both before (HR (95% CI), 1.42 (1.16-1.74), *P* < 0.001) and after PSM (HR (95% CI), 1.47 (1.14-1.89), *P* = 0.003). The survival curves showed that patients with longer APTT had a significantly lower 1-year and 4-year cumulative survival probability. The ROC of APTT combined with other severity scores significantly increased predictive ability for 1-year and 4-year mortality.

**Conclusions:**

A longer APTT (≥44) was associated with a higher risk of mortality and can serve as a prognostic predictor in CABG patients.

## 1. Introduction

Coronary artery bypass grafting (CABG) is a common procedure in cardiac surgery and a gold standard intervention in cases of severe multivessel coronary artery disease [1, 2]. For cardiac surgery, especially CABG with cardiopulmonary bypass (CPB), postoperative bleeding remains a significant source of morbidity and mortality for patients [3]. Anticoagulation therapy is involved before, during and after CABG surgery [4]. Therefore, coagulation function needs to be focused on the CABG procedure. As we known, the coagulation process is complex as a process involving multiple factors and multiple pathways [5]. Although the majority of cardiac surgical patients have no clinical evidence of bleeding diathesis, a substantial proportion may have subtle bleeding tendencies that manifest only after exposure to these hemostatically damaging effects of CPB [6]. A preoperative blood test that could accurately predict those patients who will bleed excessively after CPB would be of great practical value. Tests used for routine evaluation of the coagulation system are activated partial thromboplastin time (APTT) and international normalized ratio (INR) [7]. Previous studies have shown that early APTT is a predictor of 30-day and 1-year mortality in ST-elevation myocardial infarction patients treated with percutaneous coronary intervention and unfractionated heparin [8]. For trauma patients, APTT at admission was also a predictor of 1-year mortality [9, 10]. Therefore, we wanted to know whether the preoperative APTT as an appropriate indicator would be of prognostic value for CABG. So, using the open-source Multiparameter Intelligent Monitoring in Intensive Care III (MIMIC-III) database, we performed a retrospective study aiming to explore the impact of preoperative APTT on the prognosis of CABG-related surgery.

## 2. Methods

### 2.1. Database

The study data was extracted from a publicly available database, the Medical Information Mart for Intensive Care III (MIMIC-III) [11], comprising comprehensive and anonymous data of patients admitted to ICU of the Beth Israel Deaconess Medical Center from 2001 and 2012. Thus, the informed consent was waived by the institutional review boards (IRB). One of our authors was approved and authorized to utilize this database (Record ID: 37650993) by IRB of the Massachusetts Institute of Technology (MIT) and Beth Israel Deaconess Medical Center (BIDMC). The present study complied with the corresponding guidelines.

### 2.2. Patient Selection and Outcome

Patients in MIMIC-III who underwent CABG during this admission were collected according to ICD-9 code. Those who were younger than 18 years old or older than 89 years old, and those who were followed up for less than four years were excluded. The primary endpoint of this study was 4-year mortality, and the secondary endpoint was 1-year mortality.

### 2.3. Data Extraction

We applied pgAdmin4 based on PostgreSQL 9.6 for data management and the Structured Query Language (SQL) for data extraction. We collected the following data: baseline demographic information such as age, gender, and ethnicity; severity scores including Sequential Organ Failure Assessment (SOFA) and Simplified Acute Physical Score II (SAPS II); comorbidities including hypertension, diabetes mellitus (DM), peripheral vascular disease, myocardial infarction (MI), congestive heart failure (CHF), chronic pulmonary disease, renal failure, liver disease, and obesity. Vital signs within 24 h after ICU admission including mean systolic blood pressure (SBP), diastolic blood pressure (DBP), heart rate (HR), respiratory rate, temperature, and percutaneous oxygen saturation (SpO2) were used. The initial values of laboratory tests after ICU admission including white blood cell (WBC), hemoglobin, platelet, sodium, potassium, creatinine, glucose, and APTT were extracted. Treatments including mechanical ventilation, continuous renal replacement treatment (CRRT), and vasopressor use were also selected for analysis. As the proportion of missing values of the collected variables was less than 1%, samples with missing values were discarded in further analysis.

### 2.4. Statistical Analysis

The study population was divided into two groups according to the optimal cut-off value of APTT for 4-year mortality calculated by X-tile software. Data were summarized as medians [interquartile ranges (IQRs)] for continuous variables and number with percentages for categorical variables. Data were compared using the Mann–Whitney test for continuous variables and Pearson's *χ*^2^ test or Fisher's exact test for categorical variables appropriately.

Propensity score matching (PSM) analysis was applied to ensure the robustness of the present study. The logistic regression model was used to calculate the propensity score, in which the predefined variables included demographic information (age, gender, and ethnicity), and all variables that were statistically different at baseline (hypertension, DM, peripheral vascular disease, MI, CHF, chronic pulmonary disease, renal failure, obesity, SBP, HR, temperature, WBC, platelet, potassium, creatinine, and CRRT). Meanwhile, 1 : 1 nearest neighbor matching method was used, and the caliper width value was set as 0.02 in this study. The distribution of propensity scores for the two groups before and after matching were depicted to show common support domains, and histograms for absolute standardized differences for baseline variables before and after matching were depicted to indicate a balance.

The Kaplan-Meier curves were depicted to determine whether APTT could affect 1-year and 4-year mortality and compared by log-rank tests. Univariate and multivariable Cox proportional hazard models were used to define independent effect of higher APTT on 4-year mortality in CABG patients. Model I was adjusted for gender and age, while model II was adjusted for the variables with *P* < 0.1 in univariate Cox regression analysis. Receiver-operating characteristic (ROC) curves were depicted, and the area under the curve (AUC) was calculated to compare APTT with other severity scores. Subgroup analysis were also applied to ensure the stability of our findings in diverse subgroups, and interaction analysis were performed. All above analysis were performed using R version 4.0.3 and a two-side *P* < 0.05 was considered significant.

## 3. Results

### 3.1. Baseline Characteristics before and after PSM

After the application of selection criteria, 2706 eligible patients were included in our study cohort. The study sample was divided into two groups according to the result calculated by X-tile software: group I (APTT < 44, *n* = 2007) and group II (APTT ≥ 44, *n* = 699) (Supplement File 1). After propensity-score matching, a total of 640 patients with lower APTT were matched with 640 patients with higher APTT. The distribution of propensity scores for the two groups and the histograms for absolute standardized differences for baseline variables before and after matching indicated a good balance (Supplement Files 2 and 3). Before PSM, the baseline characteristics and significant differences of two groups were summarized in Table 1. Overall, the median age of the study patients was 68.8 (60.0-76.4) years, and approximately 26.5% of them were female. Patients with high APTT tended to be older and female (*P* values < 0.05). They had the higher prevalence of hypertension, DM, peripheral vascular disease, MI, CHF, chronic pulmonary disease, renal failure, and obesity (all *P* values < 0.05). They may have the higher values of SBP, HR, temperature, WBC, platelet, potassium, creatinine, SOFA, and SAPS II (all *P* values < 0.05). Furthermore, patients with high APTT were more likely to receive CRRT (*P* < 0.05). After PSM, the differences of variables mentioned above were balanced (Table 2).

### 3.2. Outcome of Patients before and after PSM

Before PSM, compared with APTT < 44, patients with higher APTT had longer ICU LOS and longer in-hospital mortality, 30-day mortality, 90-day mortality, 1-year mortality, and 4-year mortality (all *P* values < 0.001) (Table 3). After PSM, the similar significant differences were found in matched patients (all *P* values < 0.05) (Table 3). The results before and after matching showed that the patients with higher APTT had longer ICU LOS and higher mortality.

**Figure 1:**
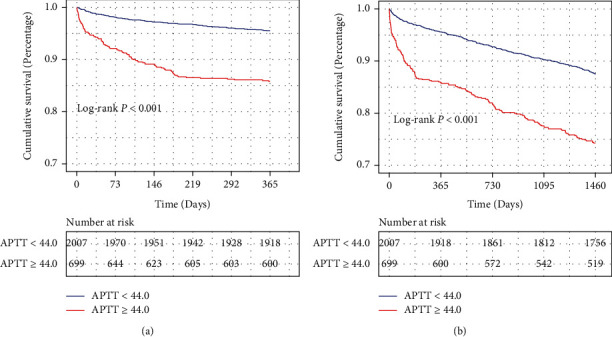
Kaplan-Meier survival analysis curves for 1-year (a) and 4-year (b) survival before propensity score matching. *P* value was calculated by log-rank test. APTT: activated partial thromboplastin time.

**Figure 2:**
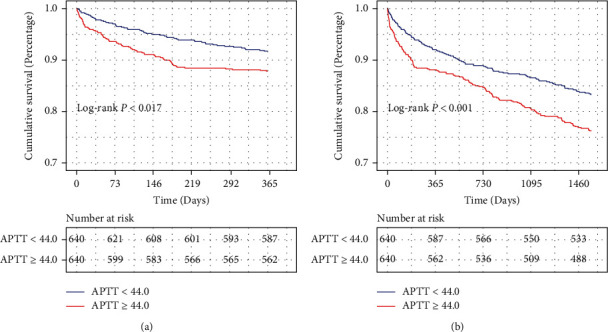
Kaplan-Meier survival analysis curves for 1-year (a) and 4-year (b) survival after propensity score matching. *P* value was calculated by log-rank test. APTT: activated partial thromboplastin time.

Kaplan-Meier analysis indicated higher mortality risk in high APTT group. Kaplan-Meier curves were used to evaluate the association between APTT level and long-term all-cause mortality. As shown in Figures 1(a) and 1(b), the survival curves showed that patients with APTT ≥ 44 had a significantly lower 1-year (log-rank test: *P* < 0.001) and 4-year (log-rank test: *P* < 0.001) cumulative survival probability compared to patients with APTT < 44. Additionally, after PSM, the survival curves (Figures 2(a) and 2(b)) indicated that the higher APTT values were still significantly associated with lower cumulative survival probability of 1 year (log-rank test: *P* = 0.017) and 4 years (log-rank test: *P* = 0.0014).

### 3.3. Cox Regression Analysis Indicated Higher Mortality Risk in High APTT Group

We fitted two Cox regression models to demonstrate the independent effects of APTT on 4-year outcome. Model 1 was adjusted for gender and age, while model 2 was adjusted for the variables with *P* < 0.1 in univariate Cox regression analysis. As shown in Table 4, compared to patients with APTT < 44, patients with APTT ≥ 44 had higher risk of 4-year all-cause death (model 1: HR (95% CI), 1.81 (1.49-2.21); *P* < 0.001; model 2: HR (95% CI), 1.42 (1.16-1.74); *P* < 0.001). After further analysis of matched cohort, a longer APTT was still regarded as an independent risk factor for 4-year all-cause mortality (model 1: HR (95% CI), 1.52 (1.19-1.95); *P* < 0.001; model 2: HR (95% CI), 1.47 (1.14-1.89); *P* = 0.003).

### 3.4. Ability of APTT to Predict 1-Year and 4-Year Mortality

**Figure 3:**
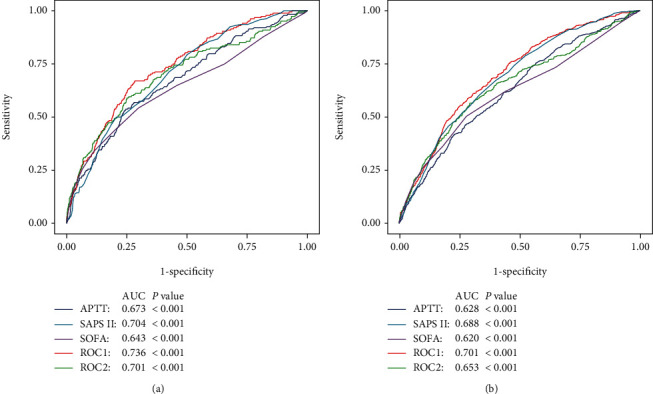
The ROC of the predictive ability of APTT for 1-year (a) and 4-year (b) mortality. ROC1 included APTT and SAPS II. ROC2 included APTT and SOFA. ROC: receiving-operating characteristic; APTT: activated partial thromboplastin time; SOFA: Sequential Organ Failure Assessment; SAPS II: Simplified Acute Physiology Score II; AUC: area under curve.

Receiver-operating characteristic (ROC) curves were depicted, and the area under the curve (AUC) was calculated to compare APTT with other severity scores. The AUCs of APTT for 1-year and 4-year mortality were only 0.673, and 0.628, respectively (Figures 3(a) and 3(b)). For 1-year mortality, the predictive ability of APTT combined with SAPS II (AUC: 0.736) was superior to SAPS II (AUC: 0.704) alone, and the significant difference was found between two groups (DeLong's test: *P* = 0.004). Meanwhile, the predictive power of APTT combined with SOFA (AUC: 0.701) was superior to SOFA (AUC: 0.643) alone with significant difference (DeLong's test: *P* < 0.001). The similar promotion of predictive ability was found for 4-year mortality, the AUC of APTT combined with SAPS II increased to 0.701 compared to SAPS II alone (ACU: 0.688; DeLong's test: *P* = 0.0023), and the AUC of APTT combined with SOFA elevated to 0.653 compared to SOFA alone (ACU: 0.620; DeLong's test: *P* < 0.001). The results indicated that the combination of APTT and traditional severity score had a good predictive value for 1-year and 4-year mortality, respectively.

### 3.5. Subgroup Analysis

Subgroup analysis was applied to ensure the stability of our findings in diverse subgroups. The patients with APTT ≥ 44 had a higher risk of 4-year death compared to those with APTT < 44 in most subgroups except for the patients who had liver disease (HR (95% CI), 3.35 (0.98-11.5); *P* = 0.054), underwent CRRT (HR (95% CI), 1.27 (0.66-2.44); *P* = 0.466), never used mechanical ventilation (HR (95% CI), 2.29 (0.96-5.44); *P* = 0.060) and vasopressor (HR (95% CI), 1.65 (0.95-2.87); *P* = 0.075) (Table 5). There was only significant interaction in CHF subgroup.

## 4. Discussion

This study explored the association between preoperative APTT and mortality among patients who underwent CABG with a 4-year follow-up. The results showed that a preoperative APTT longer than 44 seconds was a reliable predictor of 1-year and 4-year mortality. We observed for the first time the value of APTT in predicting mortality of cardiac surgery patients.

APTT, with the highest sensitivity, reflects the integrity of the intrinsic pathway of coagulation and is an index to demonstrate coagulation factor deficiency, especially in preoperative routine coagulation screening [12]. APTT is widely used to monitor the anticoagulant effect of intravenous heparin applications [13]. Therefore, for patients with CABG who use heparin for coronary heart disease before surgery, APTT has its natural advantages in evaluating preoperative coagulation function. In the present study, it was obviously observed that prolonged APTT increased the short-term and long-term mortality. Moreover, Cox-regression analysis demonstrated that APTT still showed good independent predictive value. Therefore, we believed that preoperative APTT had an important reference value for the prognosis of CABG patients.

There is no clear contraindication standard for the clinical coagulation index at present, which needs to be determined by comprehensively considering the severity of the disease, surgical model, and prognostic judgment [7]. For CABG surgery, the situation is much more complicated due to the application of preoperative anticoagulant drugs. Previous study showed that preoperative APTT was greater than 40 seconds in the group of severe bleeding after CABG [14]. Our study showed that APTT greater than 44 seconds was associated with 4-year mortality in CABG patients. Therefore, further studies are needed to determine the proper APTT value for predicting the prognosis of CABG patients. Although this APTT value could vary in different database, it could be used as a reference for prognostic analysis.

For subgroup analysis, APTT maintained its predictive capability regardless of age, gender, and most of the comorbidities. There were some results that drew our attention. Patients with obesity had a 6.17-fold higher risk of 4-year mortality with an APTT ≥ 44 s, while patients without obesity had only a 2.2-fold higher risk of 4-year mortality. Obesity is a recognized risk factor for thrombosis. Obesity makes the body in a high coagulant state affect the number of platelets and coagulation factor activity and damage the primary and secondary hemostasis ability [15–17]. If combined with prolonged APTT, the risk of bleeding increased, which could increase the risk of mortality. Though the *P* value was 0.064, which was not significant, it might be caused by the small number of people after grouping.

And we observed that a longer APTT was more associated with a bad prognosis in patients without CHF compared with patients with CHF. As such a result we thought it was owing to CHF which is an important risk factor for the prognosis of CABG [1]. CHF was one of the major adverse cardiovascular events (MACE), so in the presence of CHF the prognostic efficacy of other factors was weakened. But in the absence of CHF, a longer APTT still showed a better predictive value, for which it was reasonable to include APTT in the prediction models. Moreover, the results of ROC curve analysis showed that APTT significantly increased the AUCs when it was added to the SOFA score and SAPS II. This once again demonstrated the important value of APTT for the prognostic evaluation of CABG.

Some limitation to this study included the following: (1) the follow-up outcome could be affected by some cofounders, regarding the severity of the disease and the operative procedure; however, this database analysis was retrospective cohort study, and these situations were not recorded; (2) the study population was US adults based on MIMIC III database; thus, our results might be not applicable to different race; and (3) because this database analysis was a single center research and the sample size was relatively small, the multicenter prospective research is necessary to verify our conclusions.

## 5. Conclusions

In conclusion, we put forward that a longer APTT (≥44) was associated with a higher risk of mortality and can serve as a prognostic predictor in CABG patients. Studies of large multicenter populations are necessary for further validation.

## Figures and Tables

**Table 1:** Characteristics of the study patients before PSM.

Characteristics	Before PSM			
	Total (*n* = 2706)	APTT < 44 (*n* = 2007)	APTT ≥ 44 (*n* = 699)	*P* value
Demographics				
Age, years	68.8 (60.0-76.4)	67.5 (59.0-75.0)	72.7 (64.7-79.4)	<0.001
Gender, female	717 (26.5)	464 (23.1)	253 (36.2)	<0.001
Ethnicity, *n* (%)				0.506
White	1789 (66.1)	1336 (66.6)	453 (64.8)	
Black	71 (2.6)	51 (2.5)	20 (2.9)	
Asian	44 (1.6)	28 (1.4)	16 (2.3)	
Hispanic	44 (1.6)	31 (1.5)	13 (1.9)	
Other	758 (28.0)	561 (28.0)	197 (28.2)	
Comorbidities, *n* (%)				
Hypertension	1952 (72.1)	1472 (73.3)	480 (68.7)	0.020
DM	1010 (37.3)	793 (39.5)	217 (31.0)	<0.001
Peripheral vascular disease	355 (13.1)	243 (12.1)	112 (16.0)	0.010
MI	511 (18.9)	348 (17.3)	163 (23.3)	0.001
CHF	677 (25.0)	430 (21.4)	247 (35.3)	<0.001
Chronic pulmonary disease	356 (13.2)	246 (12.3)	110 (15.7)	0.023
Renal failure	202 (7.5)	129 (6.4)	73 (10.4)	0.001
Liver disease	39 (1.4)	24 (1.2)	15 (2.1)	0.103
Obesity	124 (4.6)	102 (5.1)	22 (3.1)	0.045
24 h vital signs				
Mean SBP, mmHg	111.4 (105.8-119.0)	111.0 (105.9-118.3)	112.8 (105.6-121.1)	0.022
Mean DBP, mmHg	56.3 (52.7-60.6)	56.3 (52.7-60.4)	56.3 (52.4-61.2)	0.597
Mean HR, beats/min	85.1 (79.2-91.1)	85.4 (79.9-91.4)	84.2 (77.3-90.1)	<0.001
Mean respiratory rate, beats/minute	16.7 (15.0-18.8)	16.8 (15.1-18.8)	16.5 (14.8-18.7)	0.051
Mean temperature, °C	36.9 (36.6-37.2)	36.9 (36.6-37.2)	36.8 (36.5-37.2)	<0.001
Mean SpO2, %	98.3 (97.3-99.0)	98.3 (97.4-99.0)	98.2 (97.3-98.9)	0.111
Laboratory parameters				
WBC, 10^9^/L	11.8 (9.1-15.3)	12.2 (9.4-15.6)	10.9 (8.3-13.8)	<0.001
Hemoglobin, g/dL	10.0 (8.9-11.2)	10.0 (9.0-11.1)	9.9 (8.7-11.3)	0.351
Platelet, 10^9^/L	156.5 (122.0-203.0)	158.0 (125.0-202.0)	152.0 (108.0-206.0)	0.002
Sodium, mmol/L	137.0 (135.0-139.0)	137.0 (135.0-139.0)	137.0 (134.0-139.0)	0.610
Potassium, mmol/L	4.3 (3.9-5.0)	4.4 (3.9-5.0)	4.2 (3.8-4.8)	0.001
Creatinine, *μ*mol/L	0.8 (0.7-1.0)	0.8 (0.7-1.0)	0.9 (0.7-1.1)	<0.001
Glucose, mg/dL	135.0 (114.0-164.0)	136.0 (114.0-164.0)	135.0 (113.0-165.0)	0.927
Management, *n* (%)				
Mechanical ventilation	2610 (96.5)	1938 (96.6)	672 (96.1)	0.686
CRRT	45 (1.7)	17 (0.8)	28 (4.0)	<0.001
Vasopressor use	2342 (86.5)	1743 (86.8)	599 (85.7)	0.481
Severity of illness, points				
SOFA	4.0 (3.0-6.0)	4.0 (3.0-6.0)	5.0 (3.0-7.0)	<0.001
SAPS II	33.0 (27.0-40.0)	32.0 (26.0-39.0)	36.0 (29.0-43.0)	<0.001
APTT, second	35.2 (30.3-44.3)	32.7 (29.1-36.5)	54.8 (47.7-69.5)	<0.001

PSM: propensity score matching; DM: diabetes mellitus; MI: myocardial infarction; CHF: congestive heart failure; SBP: systolic blood pressure; DBP: diastolic blood pressure; HR: heart rate; WBC: white blood cell; CRRT: continuous renal replacement therapy; SOFA: Sequential Organ Failure Assessment; SAPS II: Simplified Acute Physiology Score II; APTT: activated partial thromboplastin time.

**Table 2:** Characteristics of the study patients after PSM.

Characteristics	After PSM			
	Total (*n* = 1280)	APTT < 44 (*n* = 640)	APTT ≥ 44 (*n* = 640)	*P* value
Demographics				
Age, years	72.2 (64.0-78.7)	72.3 (64.2-78.7)	72.2 (63.9-78.8)	0.672
Gender, female	425 (33.2)	208 (32.5)	217 (33.9)	0.635
Ethnicity, *n* (%)				0.707
White	820 (64.1)	401 (62.7)	419 (65.5)	
Black	33 (2.6)	16 (2.5)	17 (2.7)	
Asian	24 (1.9)	11 (1.7)	13 (2.0)	
Hispanic	24 (1.9)	11 (1.7)	13 (2.0)	
Other	379 (29.6)	201 (31.4)	178 (27.8)	
Comorbidities, *n* (%)				
Hypertension	876 (68.4)	431 (67.3)	445 (69.5)	0.434
DM	403 (31.5)	199 (31.1)	204 (31.9)	0.810
Peripheral vascular disease	202 (15.8)	105 (16.4)	97 (15.2)	0.591
MI	268 (20.9)	130 (20.3)	138 (21.6)	0.631
CHF	415 (32.4)	207 (32.3)	208 (32.5)	0.999
Chronic pulmonary disease	185 (14.5)	91 (14.2)	94 (14.7)	0.874
Renal failure	123 (9.6)	60 (9.4)	63 (9.8)	0.850
Liver disease	29 (2.3)	15 (2.3)	14 (2.2)	0.999
Obesity	46 (3.6)	24 (3.8)	22 (3.4)	0.881
24 h vital signs				
Mean SBP, mmHg	112.9 (106.5-121.0)	113.1 (107.4-120.7)	112.6 (105.5-121.1)	0.132
Mean DBP, mmHg	56.2 (52.3-60.6)	56.2 (52.3-60.4)	56.1 (52.4-60.9)	0.685
Mean HR, beats/min	84.2 (78.1-90.0)	84.0 (78.3-89.9)	84.3 (77.6-90.0)	0.607
Mean respiratory rate, beats/minute	16.6 (14.9-18.8)	16.6 (15.0-18.8)	16.6 (14.8-18.7)	0.680
Mean temperature, °C	36.9 (36.6-37.2)	36.9 (36.6-37.2)	36.8 (36.6-37.2)	0.292
Mean SpO2, %	98.2 (97.3-99.0)	98.2 (97.2-99.0)	98.3 (97.3-98.9)	0.752
Laboratory parameters				
WBC, 10^9^/L	11.0 (8.4-14.1)	11.1 (8.4-14.2)	10.9 (8.3-13.9)	0.693
Hemoglobin, g/dL	9.9 (8.7-11.2)	10.0 (8.8-11.1)	9.8 (8.6-11.3)	0.830
Platelet, 10^9^/L	150.0 (114.0-201.2)	148.0 (117.0-194.0)	152.0 (110.0-206.0)	0.811
Sodium, mmol/L	137.0 (135.0-139.0)	137.0 (135.0-139.0)	137.0 (134.0-139.0)	0.908
Potassium, mmol/L	4.3 (3.9-4.9)	4.3 (3.9-4.9)	4.2 (3.8-4.9)	0.526
Creatinine, *μ*mol/L	0.8 (0.7-1.1)	0.8 (0.7-1.1)	0.9 (0.7-1.1)	0.092
Glucose, mg/dL	137.0 (115.0-165.0)	138.0 (119.0-165.2)	135.0 (113.0-164.0)	0.070
Management, *n* (%)				
Mechanical ventilation	1230 (96.1)	616 (96.2)	614 (95.9)	0.885
CRRT	26 (2.0)	11 (1.7)	15 (2.3)	0.552
Vasopressor use	1091 (85.2)	549 (85.8)	542 (84.7)	0.636
Severity of illness, points				
SOFA	5.0 (3.0-6.0)	5.0 (3.0-6.0)	5.0 (3.0-7.0)	0.591
SAPS II	35.0 (29.0-42.0)	35.0 (29.0-42.0)	35.0 (29.0-42.2)	0.796
APTT, second	44.0 (33.7-54.7)	33.7 (30.0-37.2)	54.8 (47.7-68.6)	<0.001

PSM: propensity score matching; DM: diabetes mellitus; MI: myocardial infarction; CHF: congestive heart failure; SBP: systolic blood pressure; DBP: diastolic blood pressure; HR: heart rate; WBC: white blood cell; CRRT: continuous renal replacement therapy; SOFA: Sequential Organ Failure Assessment; SAPS II: Simplified Acute Physiology Score II; APTT: activated partial thromboplastin time.

**Table 3:** Outcome of the study patients before and after PSM.

Outcomes	Total	APTT < 44	APTT ≥ 44	*P* value
Before PSM				
Number	*n* = 2706	*n* = 2007	*n* = 699	*P* value
ICU LOS (days)	2.5 (1.4-4.3)	2.2 (1.3-3.9)	3.4 (2.1-6.7)	<0.001
Mortality, *n* (%)				
In-hospital mortality	48 (1.8)	15 (0.7)	33 (4.7)	<0.001
30-day mortality	59 (2.2)	22 (1.1)	37 (5.3)	<0.001
90-day mortality	103 (3.8)	42 (2.1)	61 (8.7)	<0.001
1-year mortality	188 (6.9)	89 (4.4)	99 (14.2)	<0.001
4-year mortality	431 (15.9)	251 (12.5)	180 (25.8)	<0.001
After PSM				
Number	*n* = 1280	*n* = 640	*n* = 640	*P* value
ICU LOS (days)	3.0 (1.9-5.2)	2.3 (1.4-4.2)	3.3 (2.1-6.2)	<0.001
Mortality, *n* (%)				
In-hospital mortality	32 (2.5)	10 (1.6)	22 (3.4)	0.049
30-day mortality	37 (2.9)	11 (1.7)	26 (4.1)	0.020
90-day mortality	70 (5.5)	23 (3.6)	47 (7.3)	0.005
1-year mortality	131 (10.2)	53 (8.3)	78 (12.2)	0.027
4-year mortality	259 (20.2)	107 (16.7)	152 (23.8)	0.002

ICU LOS: length of ICU stays.

**Table 4:** Univariate and multivariate Cox regression analyses of APTT for 4-year mortality in study patients before and after PSM.

	Univariate		Multivariate			
			Model 1		Model 2	
	HR (95% CI)	*P*	HR (95% CI)	*P*	HR (95% CI)	*P*
Before PSM						
APTT	2.29 (1.89-2.77)	<0.001	1.81 (1.49-2.21)	<0.001	1.42 (1.16-1.74)	0.001
Gender	1.40 (1.14-1.71)	0.001	1.03 (0.84-1.27)	0.756	1.06 (0.86-1.30)	0.598
Age	1.07 (1.06-1.08)	<0.001	1.06 (1.05-1.07)	<0.001	1.06 (1.05-1.07)	<0.001
Hypertension	0.67 (0.55-0.81)	<0.001			0.67 (0.54-0.82)	<0.001
DM	1.12 (0.92-1.36)	0.249				
Peripheral vascular disease	1.90 (1.51-2.39)	<0.001			1.32 (1.04-1.67)	0.024
MI	1.20 (0.96-1.51)	0.113				
CHF	2.87 (2.37-3.47)	<0.001			1.80 (1.47-2.20)	<0.001
Chronic pulmonary disease	1.68 (1.32-2.13)	<0.001			1.46 (1.15-1.86)	0.002
Renal failure	2.78 (2.15-3.59)	<0.001			1.45 (1.07-1.97)	0.017
Liver disease	1.87 (1.03-3.40)	0.04			2.30 (1.26-4.21)	0.007
Obesity	0.63 (0.36-1.09)	0.101				
Mechanical ventilation	0.69 (0.45-1.08)	0.103				
CRRT	22.35 (16.04-31.14)	<0.001			13.7 (9.20-20.39)	<0.001
Vasopressor use	1.10 (0.83-1.46)	0.508				
After PSM						
APTT	1.49 (1.16-1.91)	0.002	1.52 (1.19-1.95)	0.001	1.47 (1.14-1.89)	0.003
Gender	1.14 (0.89-1.47)	0.307	1.00 (0.77-1.29)	0.971	1.03 (0.80-1.33)	0.839
Age	1.05 (1.04-1.07)	<0.001	1.05 (1.04-1.07)	<0.001	1.06 (1.04-1.07)	<0.001
Hypertension	0.75 (0.59-0.97)	0.029			0.66 (0.50-0.85)	0.002
DM	1.25 (0.97-1.61)	0.083				
Peripheral vascular disease	1.64 (1.22-2.20)	0.001			1.4 (1.04-1.89)	0.025
MI	1.06 (0.79-1.42)	0.691				
CHF	2.31 (1.81-2.94)	<0.001			1.77 (1.37-2.28)	<0.001
Chronic pulmonary disease	1.35 (0.99-1.85)	0.061			1.21 (0.88-1.66)	0.248
Renal failure	2.33 (1.69-3.21)	<0.001			1.53 (1.06-2.21)	0.025
Liver disease	1.42 (0.70-2.87)	0.327			1.98 (0.97-4.04)	0.059
Obesity	1.07 (0.57-2.02)	0.825				
Mechanical ventilation	0.57 (0.35-0.95)	0.031				
CRRT	15.54 (10.06-23.99)	<0.001			14.51 (8.82-23.89)	<0.001
Vasopressor use	1.22 (0.85-1.76)	0.279				

**Table 5:** Subgroup analysis for the effect of APTT on 4-year mortality in study patients before PSM.

Characteristics	*N* (%)	APTT ≥ 44		*P* value for interaction
		HR (95% CI)	*P* value	
Age				0.084
≤75	1911 (70.62)	2.34 (1.76-3.11)	<0.001	
>75	795 (29.38)	1.67 (1.29-2.17)	<0.001	
Gender				0.990
Female	717 (26.50)	2.22 (1.59-3.08)	<0.001	
Male	1989 (73.50)	2.22 (1.75-2.82)	<0.001	
Hypertension				0.997
No	754 (27.86)	2.23 (1.62-3.07)	<0.001	
Yes	1952 (72.14)	2.27 (1.78-2.88)	<0.001	
DM				0.620
No	1696 (62.68)	2.24 (1.75-2.86)	<0.001	
Yes	1010 (37.32)	2.49 (1.83-3.4)	<0.001	
Peripheral vascular disease				0.486
No	2351 (86.88)	2.17 (1.74-2.69)	<0.001	
Yes	355 (13.12)	2.55 (1.69-3.85)	<0.001	
MI				0.142
No	2195 (81.12)	2.11 (1.69-2.62)	<0.001	
Yes	511 (18.88)	2.96 (1.97-4.45)	<0.001	
CHF				0.019
No	2029 (74.98)	2.48 (1.91-3.23)	<0.001	
Yes	677 (25.02)	1.56 (1.18-2.06)	0.002	
Chronic pulmonary disease				0.076
No	2350 (86.84)	2.45 (1.98-3.03)	<0.001	
Yes	356 (13.16)	1.58 (1.02-2.44)	0.042	
Renal failure				0.239
No	2504 (92.54)	2.1 (1.7-2.59)	<0.001	
Yes	202 (7.46)	2.82 (1.76-4.53)	<0.001	
Liver disease				0.561
No	2667 (98.56)	2.25 (1.86-2.74)	<0.001	
Yes	39 (1.44)	3.35 (0.98-11.5)	0.054	
Obesity				0.064
No	2582 (95.42)	2.2 (1.81-2.68)	<0.001	
Yes	124 (4.58)	6.17 (2.07-18.36)	0.001	
Mechanical ventilation				0.981
No	96 (3.55)	2.29 (0.96-5.44)	0.060	
Yes	2610 (96.45)	2.29 (1.88-2.78)	<0.001	
CRRT				0.318
No	2661 (98.34)	2.1 (1.71-2.57)	<0.001	
Yes	45 (1.66)	1.27 (0.66-2.44)	0.466	
Vasopressor use				0.204
No	364 (13.45)	1.65 (0.95-2.87)	0.075	
Yes	2342 (86.55)	2.4 (1.96-2.95)	<0.001	
SOFA				0.479
≤7	2354 (86.99)	2.01 (1.6-2.53)	<0.001	
>7	352 (13.01)	2.29 (1.59-3.31)	<0.001	
SAPS II				0.257
≤47	2371 (87.62)	2.08 (1.67-2.6)	<0.001	
>47	335 (12.38)	2.63 (1.78-3.88)	<0.001	

## Data Availability

The data used in the study was extracted from MIMIC III database.

## Supplementary Materials

Supplementary 1 Supplement file 1: the visual output of X-tile software for the optimal cut-off value of APTT (44 seconds) for 4-year mortality.

Supplementary 2 Supplement file 2: the distribution of propensity scores for the two groups before and after matching.

Supplementary 3 Supplement file 3: the histograms of propensity scores for the two groups before and after matching.
