# Supplementary material for: Activated Partial Thromboplastin Time and Mortality in Coronary Artery Bypass Grafting Patients
Source: Dis Markers. 2022 Sep 17;2022:2918654. doi: 10.1155/2022/2918654 (PMC9509521; doi:10.1155/2022/2918654)

Statistics

Pt No	% Total	Events	Rate	Rank	Range
2007	74.17	251	12.51	0 to 218	19.30 thru 43.90
699	25.83	180	25.75	219 to 542	44.00 thru 150.00
2706	100.00	431	15.93	0 to 542	19.30 thru 150.00

Miller-Seigmund P	<0.0001	Max: <0.0001
Chi-sq Hi/Lo	75.9987	Max: 75.9987
Relative Risk 1 vs 2	1.00 / 2.06	

Survival Analysis: studyvar : Chi-sq Hi/Lo

Setup Type Refresh

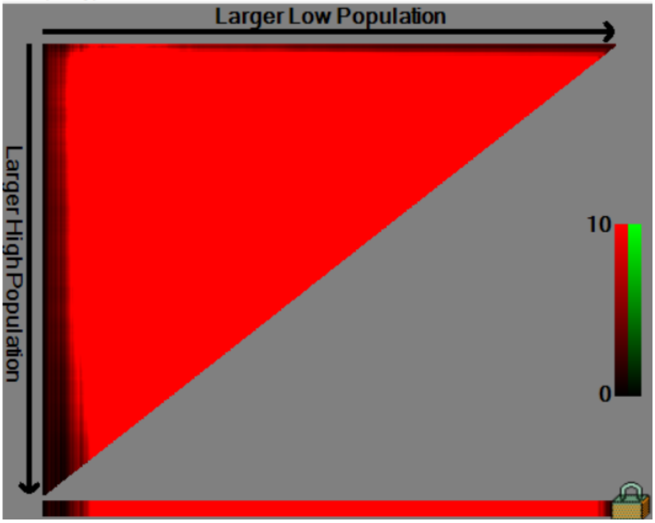

Histogram

Options

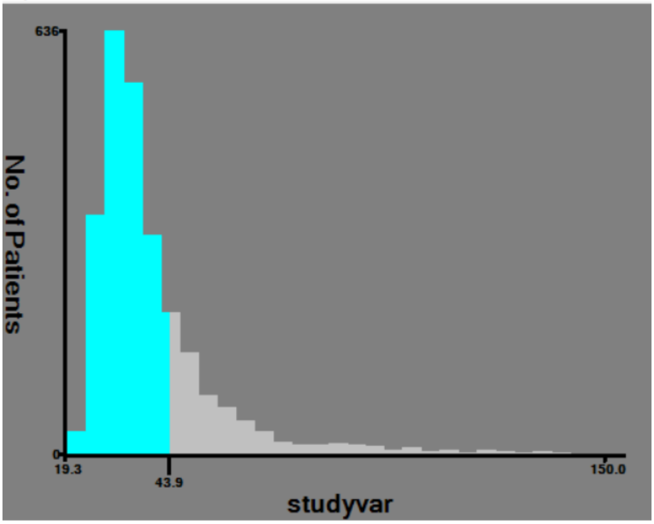

Kaplan-Meier Analysis

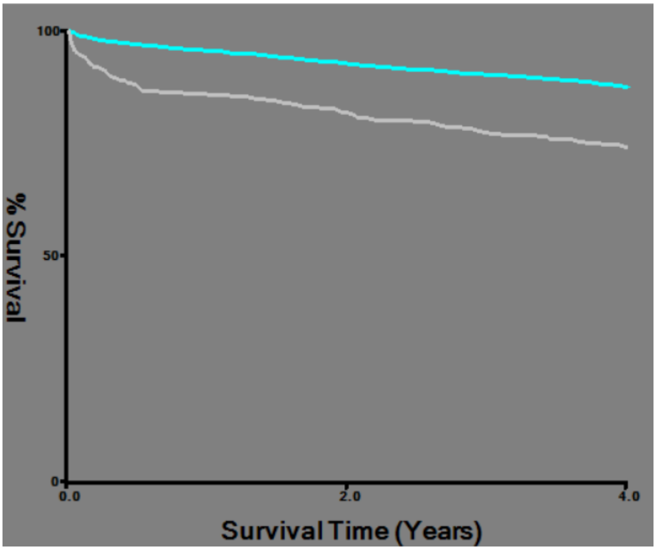

Supplement: Supplementary 1 — Supplement file 1: the visual output of X-tile software for the optimal cut-off value of APTT (44 seconds) for 4-year mortality. [file 2918654.f1.pdf]
