# Supplementary figures and images for: Activated Partial Thromboplastin Time and Mortality in Coronary Artery Bypass Grafting Patients
Source: Dis Markers. 2022 Sep 17;2022:2918654. doi: 10.1155/2022/2918654 (PMC9509521; doi:10.1155/2022/2918654)

Distribution of Propensity Scores

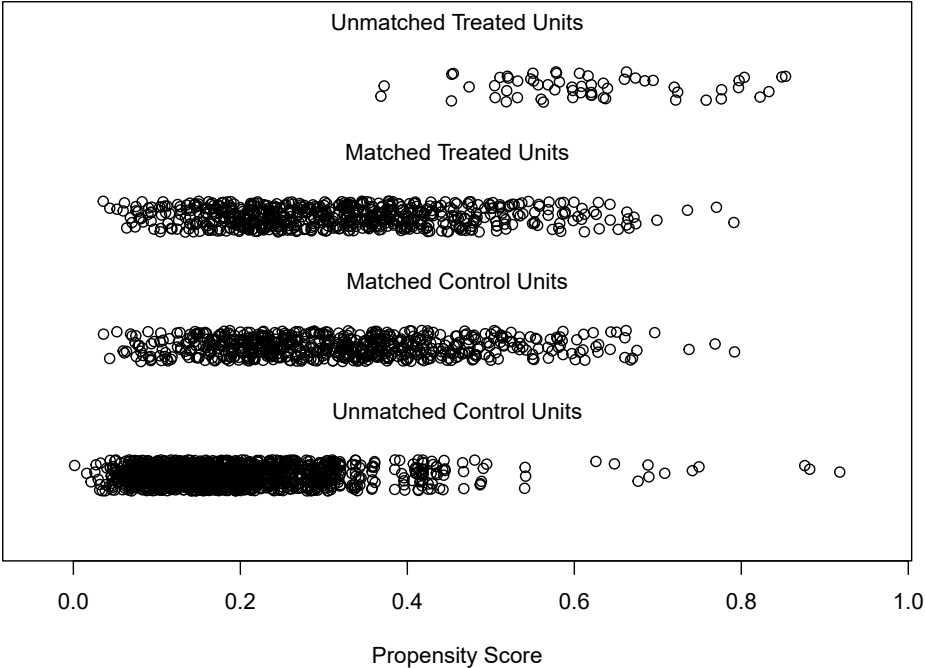

Supplement: Supplementary 2 — Supplement file 2: the distribution of propensity scores for the two groups before and after matching. [file 2918654.f2.pdf]

**Raw Treated**

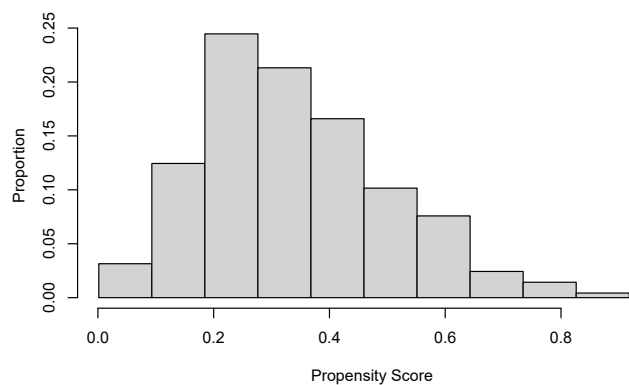

**Matched Treated**

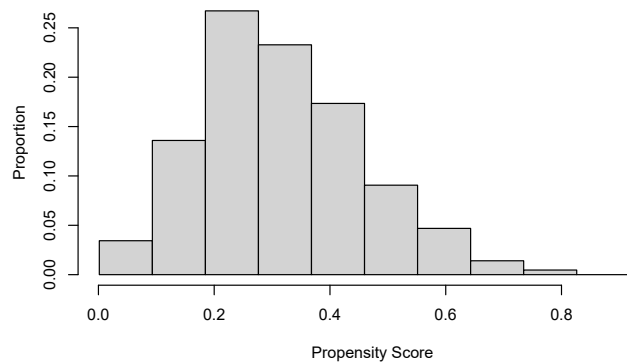

**Raw Control**

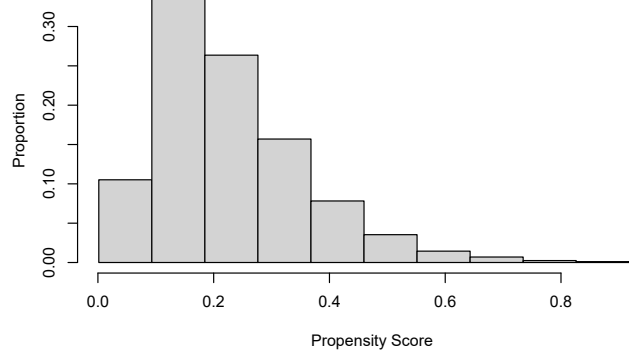

**Matched Control**

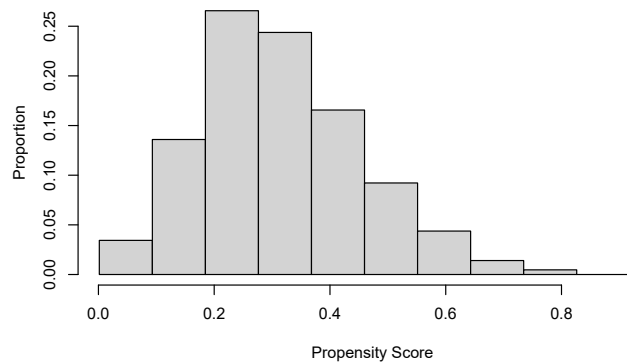

Supplement: Supplementary 3 — Supplement file 3: the histograms of propensity scores for the two groups before and after matching. [file 2918654.f3.pdf]
